# Fetal Midgut Volvulus with a Cystic Appearance, Accompanying a Sinus Rhythm and an Increased Peak Systolic Velocity without Anemia

**DOI:** 10.1155/2015/354619

**Published:** 2015-12-08

**Authors:** Metin Kaba, Aysegul Oksuzoglu, Gokcen Kaba, Hakan Timur, Eren Akbaba, Kadriye Turgut

**Affiliations:** ^1^Department of Obstetrics and Gynecology, Zekai Tahir Burak Women's Health Education and Research Hospital, Ankara, Turkey; ^2^Department of Internal Medicine, Ankara Education and Research Hospital, Ankara, Turkey

## Abstract

A midgut volvulus rarely occurs in a fetus; however, when it does, it requires an immediate diagnosis and surgery. Thirty-week pregnant was referred to our clinic with a diagnosis of a fetal abdominal cystic mass and preterm labor. The initial ultrasound examination revealed a female fetus with a 55 × 50 mm cystic mass in the lower abdomen, which was preliminarily diagnosed as an ovarian cyst. There was a sinusoidal rhythm on cardiography. The middle cerebral artery peak systolic velocity was 60.4 cm/sec, compatible with 1.49 MoMs that suggested fetal anemia on Doppler examination. Uterine contractions were observed with tocography and maternal hydration was administered for tocolytic treatment. Despite hydration, uterine contractions continued and the infant was delivered. A newborn ultrasonographic evaluation revealed a 6 cm abdominal cyst, and plain abdominal radiographs revealed distended loops of the small bowel on the left side. Emergency surgery was performed. A midgut volvulus leading to dilatation and necrosis of the small bowel without anatomical causes was observed during laparotomy. The necrotic bowel loop was resected and an end-to-end anastomosis was performed. The newborn died due to multiorgan failure. Obstetricians should be familiar with the appropriate diagnosis and management of a fetal volvulus.

## 1. Introduction

A midgut volvulus is defined as the twisting of the small bowel or proximal colon around the superior mesenteric artery or its branches, leading to an intestinal obstruction and infarction that are life-threatening conditions [1, 2]. Thus, a quick diagnosis and immediate intervention are required to decrease morbidity and mortality [1]. A midgut volvulus can occur during both the prenatal and postnatal periods. A postnatal midgut volvulus usually occurs due to malrotation of the bowel. However, a fetal midgut volvulus is a very rare condition that may occur without malrotation [2, 3]. A midgut volvulus without malrotation may not have the usual signs of a volvulus with malrotation. Thus, a prenatal diagnosis of a volvulus demands special consideration.

## 2. Case Report

**Figure 1:**
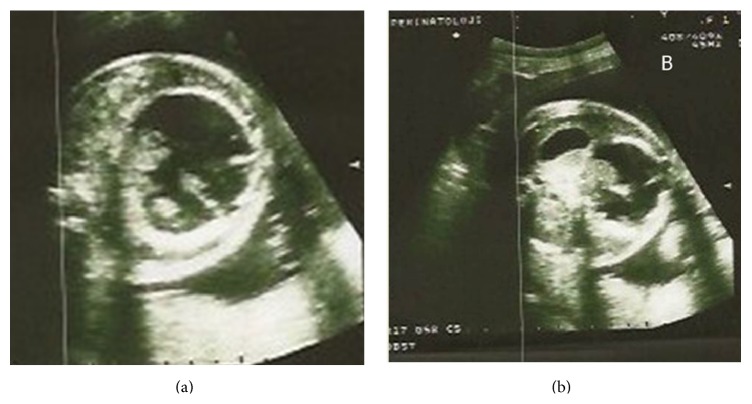
(a) A thick-walled cystic mass with papillary projections in the fetal abdomen. (b) A Fetal abdominal cyst and the fetal stomach.

**Figure 2:**
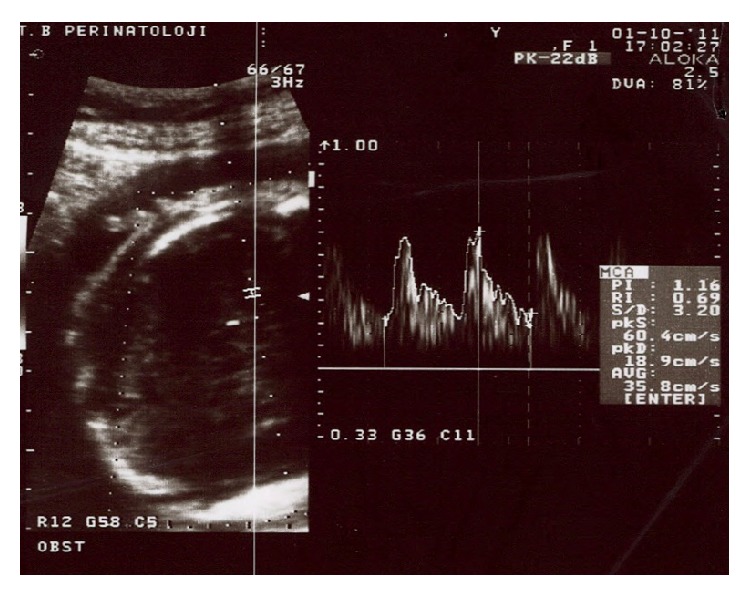
Increased peak systolic velocity in the middle cerebral artery measured at 60.4 cm/sec with 1.49 MoMs, which was suggestive of fetal anemia.

A 33-year-old, gravida 2, para 1, 30-week pregnant woman was referred to our clinic with a diagnosis of an abdominal cystic mass in a female fetus and threatening preterm labor. The fetal chromosome analysis was normal, which had been performed due to a high risk for Down syndrome in the triple test. The obstetrics examination revealed 3 cm cervical dilatation with 70% effacement, vertex presentation, and an intact amniotic membrane. The ultrasonographic evaluation revealed a female fetus in accordance with 30 weeks of gestation and a 55 × 50 mm cystic mass in the lower abdomen without ascites and polyhydramnios. The cyst had a thick wall and papillary projections into the lumen, which led to a preliminary diagnosis of an ovarian mass (Figure 1(a)). The fetal stomach (Figure 1(b)) and vesica urinaria were observed separately. The peak systolic velocity in the middle cerebral artery was 60.4 cm/sec, compatible with 1.49 MoMs for 30-week gestation on Doppler examination, which suggests fetal anemia (Figure 2).

**Figure 3:**
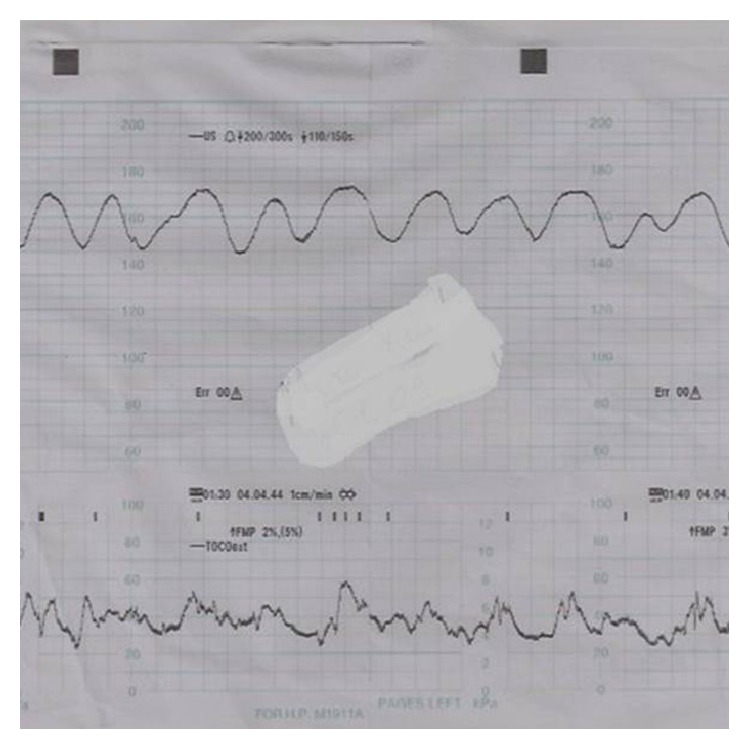
Fetal cardiac sinus rhythm.

The patient was admitted to the high risk prenatal care unit with a diagnosis of threatened preterm delivery and fetal abdominal cystic mass. Maternal hydration and steroids were administered to prevent preterm delivery and to accelerate fetal lung maturation, respectively. There was a severe sinus rhythm on cardiography that continued until delivery (Figure 3). Despite hydration, the frequency and severity of uterine contractions progressed and the fetus was delivered transvaginally at 9 hours of admission. The newborn was a 1,625 g female. Apgar scores were 1 and 3 at 1 and 5 minutes, respectively. The newborn was resuscitated with endotracheal intubation in the delivery room and transported to the neonatal intensive care unit where she was monitored with continuous positive airway pressure. A physical examination revealed prominent abdominal distention. The complete blood count assessment revealed white blood cells: 27.800/mm^3^; platelets: 129,000/mm^3^; hemoglobin (Hb): 14 mg/dL; and hematocrit: 42%. The umbilical artery blood gas assessment revealed pH: 7.14; PO_2_: 51.3 mmHg; and PCO_2_: 60.44 mmHg. A plain abdominal radiography revealed a markedly distended abdomen and distended loops of small bowel on the left side. Abdominal ultrasonography revealed a 60 cm diameter cystic mass extending from the epigastrium to the pelvis. The neonatal condition worsened and an explorative laparotomy was performed with the diagnosis of an abdominal mass at postnatal 48 hours. The laparotomic exploration revealed a midgut volvulus without malrotation that led to dilatation and necrosis of the small bowel. The twisted bowel loop became a necrotized cystic mass approximately 7 cm in diameter that was located 20 cm proximal to the ileocecal valve. The necrotic bowel loop was resected and an end-to-end anastomosis was performed. Unfortunately, the newborn died at the postoperative fourth hour due to multiorgan failure.

## 3. Discussion

Here we presented a case of midgut volvulus without malrotation and the usual signs that led to a misdiagnosis of an ovarian mass. A prenatal diagnosis of a midgut volvulus may be difficult if the classic signs are not observed on ultrasonographic evaluations. A fetal midgut volvulus can be diagnosed easily when an ultrasound examination reveals dilated loops of bowel, whirlpool signs, polyhydramnios, ascites, and signs of anemia such as increased peak systolic velocity in the middle cerebral artery and sinus rhythm [4]. If the volvulus leads to intestinal necrosis and perforation, hemorrhagic ascites, peritoneal calcification, and a pseudocyst may develop that could be observed on an ultrasonographic examination [5]. Sequestration of blood from the necrotized intestine can cause fetal ascites and anemia [6]. A fetal cardiac sinus rhythm may be observed in the fetus with anemia and hypoxia/ischemia that leads to fetal distress [4]. Observation of dilated bowel loops and fetal ascites accompanied by an increased peak systolic velocity in the middle cerebral artery suggesting fetal anemia on an ultrasonographic evaluation are indicative of a fetal midgut volvulus [7]. In our case, there were no classic suggestive signs of a midgut volvulus such as whirlpool signs, ascites, and polyhydramnios. Alternatively, there was an increased peak systolic velocity in the middle cerebral artery and a cardiac sinus rhythm, both of which were signs of fetal distress.

A midgut volvulus can lead to ischemic necrosis that causes fetal distress, which might activate the release of stress hormones that lead to an activation of uterine contractions and preterm delivery [8]. The main complaint of a pregnant woman with a fetal midgut volvulus is the decrease of fetal movement. Nonstress tests can show different stages of fetal distress, such as late decelerations and poor variability [9]. In our case, the presence of a fetal cardiac sinus rhythm, an increased peak systolic velocity in the middle cerebral artery, and a threatened preterm labor could be signs of fetal distress.

A fetal midgut volvulus is commonly associated with intestinal malrotation or congenital anomalies such as omphalocele, gastroschisis, intestinal atresia, or an annular pancreas. On the other hand, the etiology of a volvulus without malrotation is unknown, and associated anomalies are rare [8]. The absence of a small bowel muscle segment or a mesenteric defect might be associated with a fetal midgut volvulus without malrotation [10]. Recently, Kargl et al. classified volvuli into three groups, a “classical volvulus” (associated with malposition), a “segmental volvulus” (causative anatomic anomaly), and a “volvulus without malrotation,” according to their clinical and radiological presentation and the outcome of treatment [11]. They also observed that a remarkable volvulus without a causative anatomic anomaly has been detected in preterm infants. Therefore, they suggest that a volvulus without malrotation has to be recognized as a distinct clinical and pathological condition in this age group. The affected bowel segment is usually small in a volvulus without malrotation. Although ischemic bowel damage occurs more rapidly, the remaining small intestine segment is generally long enough to provide sufficient enteral nutrition. Despite the difficulties in early diagnosis of a volvulus without malrotation, the outcome of treatment seems to be better than with the classical volvulus due to the smaller portion of the affected bowel [11]. According to the new classification, our case was a volvulus without malrotation with the absence of any classic signs of a volvulus. A volvulus without malrotation particularly affects very low birth weight and extremely low birth weight infants and may lead to fetal distress and preterm delivery, as in our case. At birth, abdominal distension, bilious vomiting, and failure of meconium passage are the presenting symptoms of a volvulus in a newborn [12]. Dilated bowel loops are often visualized on plain abdominal X-ray films, as in our case [12].

When a midgut volvulus is detected in a fetus, emergency cesarean section and surgical intervention can reduce the morbidity and mortality [13]. The prognosis depends on birth weight, gestational age, level of prematurity, the length of the affected bowel, and associated anomalies [13]. An appropriate timing of delivery, timely diagnosis of the volvulus, and emergency surgical intervention could be important for decreasing fetal morbidity and mortality.

## 4. Conclusion

A prenatal midgut volvulus without malrotation is quite a rare condition and the diagnosis is very difficult without the usual classical volvulus signs. A midgut volvulus requires an early diagnosis and immediate intervention following delivery. Thus, an accurate diagnosis and a multidisciplinary team approach are required. If a cystic mass is detected in the lower fetal abdomen with fetal distress signs such as sinus rhythm and increased peak systolic velocity in the middle cerebral artery, a midgut volvulus should be considered, and the pregnant woman should be referred to a tertiary center.
